# Clinical Validation of MyProstateScore 2.0 Testing Using First-Catch, Non-DRE Urine

**DOI:** 10.1097/JU.0000000000004421

**Published:** 2025-01-21

**Authors:** Jeffrey J. Tosoian, Yuping Zhang, Jacob I. Meyers, Spencer Heaton, Javed Siddiqui, Lanbo Xiao, Keavash D. Assani, Daniel A. Barocas, Ashley E. Ross, Zoey Chopra, Grace C. Herron, Jacob A. Edelson, Nathan J. Graham, Udit Singhal, Simpa S. Salami, Todd M. Morgan, Ganesh S. Palapattu, John T. Wei, Arul M. Chinnaiyan

**Affiliations:** 1 Department of Urology, Vanderbilt University Medical Center, Nashville, TN; 2 Vanderbilt-Ingram Cancer Center, Nashville, TN; 3 Department of Pathology, University of Michigan, Ann Arbor, MI; 4 Lynx Dx, Ann Arbor, MI; 5 Department of Urology, Northwestern University Feinberg School of Medicine, Chicago, IL; 6 Department of Urology, University of Michigan, Ann Arbor, MI; 7 Division of Urology, Department of Surgery, University of Chicago Medicine, Chicago, IL; 8 Rogel Cancer Center, Ann Arbor, MI; 9 Howard Hughes Medical Institute, Chevy Chase, MD

**Keywords:** biomarkers, early detection of cancer, prostate cancer, liquid biopsy

## Abstract

**Purpose:**

The 18-gene MyProstateScore 2.0 (MPS2) test was previously validated for detection of Grade Group≥2 (GG≥2) prostate cancer using post-digital rectal examination (DRE) urine. To improve ease of testing, we validated MPS2 using first-catch, non-DRE urine.

**Materials and Methods:**

Patients provided first-catch urine prior to biopsy. MPS2 values were calculated using previously-validated models differing only by extent of clinical data included: biomarkers alone (BA; no clinical data), biomarkers and clinical factors (BA+CF), and biomarkers, clinical factors, and prostate volume (BA+CF+PV). The primary outcome was GG≥2 cancer on biopsy. MPS2 performance and clinical consequences of testing were compared to PSA and the Prostate Cancer Prevention Trial risk calculator (PCPTrc).

**Results:**

The cohort included 266 men with median PSA 6.6 ng/mL (IQR 4.9-9.1), of which 103 (39%) had GG≥2 cancer on biopsy. The area under the curve for GG≥2 cancer was 57% for PSA, 62% for PCPTrc, and 71%, 74%, and 77% for MPS2 models. Under a testing approach detecting >90% of GG≥2 cancers, MPS2 testing would have avoided 36-42% of unnecessary biopsies, as compared to 13% using the PCPTrc. In patients with a prior negative biopsy, MPS2 testing would have avoided 44-53% of repeat biopsies, as compared to only 2.6% using PCPTrc.

**Conclusions:**

Using first-catch urine, MPS2 meaningfully improved the proportion of biopsies avoided relative to PCPTrc while maintaining highly-sensitive detection of GG≥2 cancer. Non-DRE testing provides a convenient, objective, and highly-accurate testing option to reduce the need for imaging and biopsy in men with elevated PSA.

## INTRODUCTION

Screening with serum prostate-specific antigen (PSA) has been shown to significantly reduce prostate cancer (PCa) mortality.^1^ Long-term follow-up from the Goteborg randomized screening trial revealed a number needed to invite to screen of 221 patients to prevent one PCa death,^1^ comparing favorably to other prevalent cancers.^2^ At the same time, the traditional approach to PSA screening – in which patients with an elevated serum PSA are directed to prostate biopsy – is associated with potential harms, including unnecessary biopsies and overdiagnosis of low-grade, indolent cancers.^3^ As such, contemporary guidelines emphasize a focus on higher-grade, clinically-significant prostate cancers (Grade Group≥2 [GG≥2]) through the use of magnetic resonance imaging (MRI) or biomarker testing prior to biopsy.^4-5^

Indeed, prostate MRI followed by targeted biopsy of abnormal regions has proven effective in detecting GG≥2 cancer and reducing overdiagnosis of GG1 disease under some diagnostic pathways.^6^ However MRI is resource dependent, and its population-wide use is limited by availability, dependence on expert interpretation, and, in the United States, cost.^7-9^ As a result, there is great interest in the use of blood- and urine-based biomarkers in men with elevated PSA to rule out benign and low-grade lesions, preserving the use of MRI and biopsy for higher-risk men most likely to benefit.^10^ Several blood-based and urine-based biomarker tests are available to that end.^11^

One such option is the urinary MyProstateScore 2.0 (MPS2) test. Using urine samples obtained after digital rectal examination (DRE), the assay measures expression of 18 cancer-specific and high-grade cancer-specific genes to provide an individualized risk of GG≥2 cancer.^12^ On external validation, MPS2 testing reduced unnecessary biopsies by 35%-51% while maintaining detection of 95% of GG≥2 cancers. Yet DRE is uncomfortable for patients and is now considered optional by clinical guidelines.^4-5^ Moreover, performing DRE is not feasible in the growing population undergoing telehealth consultations. As such, in the current study, we validated the MPS2 test in urine specimens obtained without DRE and evaluated its clinical performance for detection of GG≥2 PCa.

## MATERIALS AND METHODS

### Study Population and Protocol

Approval was obtained from the University of Michigan (U-M) Institutional Review Board (HUM00042749), and all participants provided written informed consent. The study population included patients without PCa that underwent prostate biopsy at U-M from November 2020 to March 2023 for elevated PSA and/or abnormal DRE. Initial biopsy patients with PSA>20 ng/ml (n=7) and repeat biopsy patients with PSA>25 ng/ml (n=0) were excluded.^5^ All patients underwent 12-core systematic biopsy via transrectal or transperineal approach. Based on clinical judgment, a proportion of patients underwent pre-biopsy MRI. Patients with suspicious lesions (defined as Prostate Imaging Reporting and Data System≥3 [PI-RADS≥3]) underwent targeted biopsy of the lesions.

Participants provided ≤40 mL of first-catch urine without prior DRE. Per institutional protocol, specimens were stored unbuffered at −80°C until processing. RNA extraction was performed from ≤5 mL of urine using an extraction method adapted and optimized for non-DRE urine. RNA was reverse transcribed to cDNA and biomarker amplification and measurement were performed using the QuantStudio^™^ 12K Flex Real-Time PCR System (Thermo Fisher Scientific) as described.^12^ Expression was quantified by the relative cycle threshold (Crt), defined as the number of amplification cycles required for fluorescence to exceed background level. All samples were run in triplicate, and mean Crt values were normalized to the housekeeping gene KLK3. Normalized mean Crt values were used to calculate risk scores using the previously-validated MPS2 models.^12^

To maximize predictive value for each patient based on the extent of clinical data available, all three previously-established MPS2 models were validated: i) a model using biomarkers alone (BA; no clinical data included), ii) a model including biomarkers and clinical factors (BA+CF; age, race, PSA, DRE findings, family history, prior negative biopsy status), and iii) a model including biomarkers, clinical factors, and prostate volume (BA+CF+PV). Notably, addition of clinical factors did not improve performance in the repeat biopsy population, thus the MPS2(BA) model is used when standard clinical factors without volume are provided in this setting. The assay measures RNA expression of 18 genes: 4 high-grade cancer–specific genes (*APOC1*, *B3GNT6*, *NKAIN1*, *SCHLAP1*), 13 cancer-specific genes (*PCGEM1*, *SPON2*, *TRGV9*, *PCA3*, *OR51E2*, *CAMKK2*, *TFF3*, *PCAT14*, *TMSB15A, HOXC6*, *ERG*, *TMPRSS2:ERG*, and *KLK4*), and the reference gene *KLK3*.^12^ MPS2 results are standardized to represent the percentage likelihood of detecting GG≥2 cancer (0%-100%) on biopsy.

### Statistical Analysis

The primary outcome was detection of GG≥2 cancer on biopsy. GG≥3 cancer was evaluated secondarily. Performance of the non-DRE MPS2 assay for GG≥2 cancer was compared to logistic regression models for serum PSA and the Prostate Cancer Prevention Trial risk calculator (PCPTrc), which builds upon the PSA-only model by also including age, race, family history of prostate cancer, DRE findings, and previous negative biopsy status.^13^ Overall discriminative performance was quantified as the area under the curve (AUC), and comparisons were performed using the Delong method.^14^ Decision curve analysis (DCA) quantified the net benefit of biomarker testing on the decision for biopsy compared to: i) performing biopsy in all patients and ii) performing biopsy in no patients.^15^ Because an estimated risk of GG≥2 cancer less than 5% justifies foregoing biopsy and a risk greater than 20% justifies performing biopsy in most patients, DCA included threshold probabilities spanning this range (0%-30%). DCA was performed using dca in the R package dcurves.^16^ Statistical analyses were performed using R version 4.1.1.

### Threshold Analysis

The MPS2 test provides a continuous percentage risk of GG≥2 cancer to best support individualized decision-making. In light of the strong motivation in medicine to provide thresholds to support decision-making,^17-18^ we secondarily sought to establish a testing threshold (i.e. cutoff) to broadly identify patients at the lowest risk of harboring GG≥2 cancer. Threshold identification was based primarily on clinical considerations. Considering the relative harms of false-positive testing (i.e. unnecessary MRI or biopsy) and false-negative testing (i.e. missed detection of GG≥2 cancer), and in alignment with the proposed role of biomarkers for rule-out testing, we sought a threshold providing a low (≤10%) false-negative rate.^19-20^ Candidate values spanning this false negative rate (i.e. 0-15%) were evaluated, and the value providing an optimal balance of biopsy avoidance and reliable rule-out ability was evaluated. Test performance and clinical consequences of testing were calculated for MPS2 and PCPTrc at a projected GG≥2 prevalence of 25%, consistent with prior analyses and published biomarker populations (17%-31%).^21-25^ To ensure level comparison, the PCPTrc threshold yielding a false negative rate equivalent to MPS2(BA) for each subgroup was evaluated. Finally, we performed subgroup analysis in patients with PSA<10 ng/ml and evaluated MPS2 and MRI in patients that underwent pre-biopsy MRI.

## RESULTS

### Study Cohort

The study cohort included 266 men of median age 67 years (IQR 62-71) and median PSA 6.6 ng/mL (IQR 4.9-9.1) (Table 1). Fifty-four men (20%) had undergone a previous negative biopsy, and 47 (18%) underwent pre-biopsy MRI, of which 25 (53%) had a PI-RADS ≥3 lesion and underwent targeted sampling. Overall, GG≥2 cancer was detected in 103 men (39%), of which 83 had GG2 (31%), 9 had GG3 (3.4%), and 11 had GG4-5 disease (4.1%).

Median PSA did not significantly differ between patients with and without GG≥2 cancer (6.8 vs. 6.4 ng/ml, p=0.06). Median MPS2 values were significantly higher in patients with versus without GG≥2 cancer across all models: 31% vs. 15% using MPS2(BA), 36% vs. 15% using MPS2(BA+CF), and 41% vs. 13% using MPS2(BA+CF+PV) (all p<0.001). Notably, the proportion of patients that underwent MRI did not significantly differ between those with and without GG≥2 cancer (18% vs. 17%, p>0.9).

### MPS2 Comparative Performance

As compared to PSA (AUC 57%, 95% CI 50%-64%) and the PCPT risk calculator (AUC 62%, 95% CI 55%-69%), all MPS2 models provided improved AUC, spanning 71% (95% CI 65%-77%) for MPS2(BA), 74% (95% CI 68%-80%) for MPS2(BA+CF), and 77% (95% CI 71%-83%) for MPS2(BA+CF+PV). All AUC comparisons were statistically significant except MPS2(BA) versus the PCPTrc, which did not meet conventional levels of significance (p=0.054, Table 2). Similar relationships were observed on subgroup analyses by previous biopsy status (Supplementary Figures 1-2). While MPS2(BA+CF) provided 3% improvement in AUC relative to MPS2(BA), this did not meet conventional levels of statistical significance (p=0.076). MPS2(BA+CF+PV) provided statistically significant improvement in AUC relative to MPS2(BA) and MPS2(BA+CF) models (Table 2).

**Figure 1.**
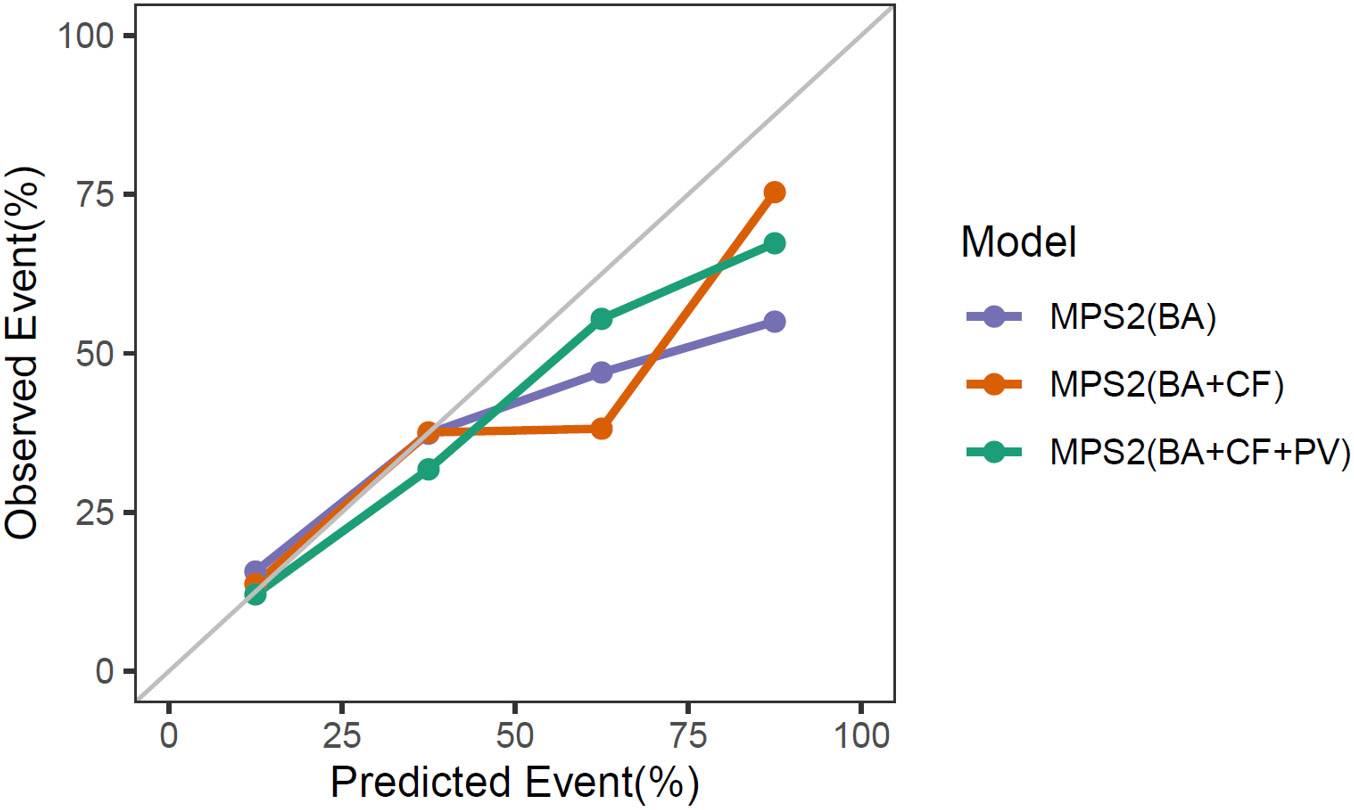
Calibration curves for GG≥2 cancer for MPS2 models in the validation cohort. BA, Biomarkers Alone Model; BA+CF, Biomarkers plus Clinical Factors Model; BA+CF+PV, Biomarkers, Clinical Factors, and Prostate Volume Model.

**Figure 2.**
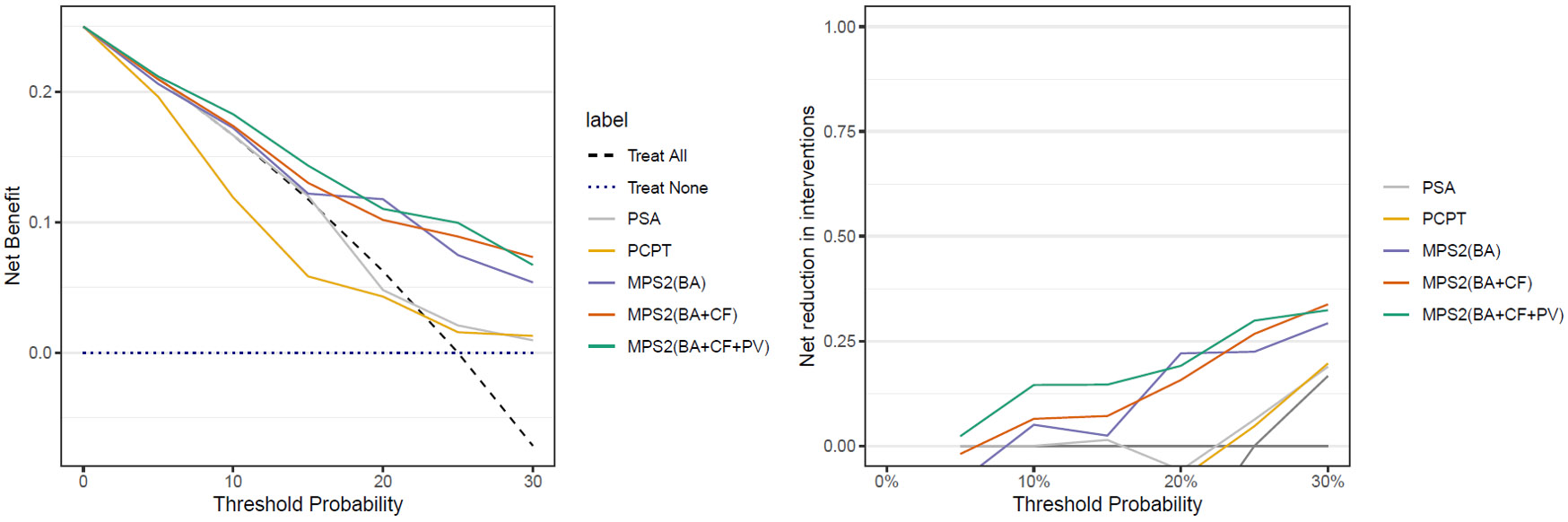
Decision curve analysis (DCA) plots for the outcome of GG≥2 cancer based on pre-biopsy testing with PSA, the PCPT risk calculator, and MPS2 models compared to baseline approaches of biopsying all patients and biopsying no patients. BA, Biomarkers Alone Model; BA+CF, Biomarkers plus Clinical Factors Model; BA+CF+PV, Biomarkers, Clinical Factors, and Prostate Volume Model. **(A)** Net clinical benefit, in which the unit of net benefit (y-axis) is true positives. A net benefit of 0.1 is equivalent to an approach in which an additional 10 patients per 100 are directed to biopsy, and all 10 patients are found to have GG≥2 cancer. **(B)** Net reduction in biopsies, in which the y-axis represents the net reduction in biopsies performed per 100 patients without missing a single diagnosis of GG≥2 cancer.

MPS2-based predicted probabilities of GG≥2 cancer closely approximated observed prevalence, reflecting good calibration in the study population (Figure 1) and upon calibration at 25% prevalence (Supplementary Figure 3).^21-25^ On DCA, MPS2 models provided higher net clinical benefit and a higher net reduction in biopsies per 100 patients than PSA and the PCPT risk calculator over clinically-pertinent threshold probabilities (Figure 2). Similar findings were observed in the biopsy-naïve subpopulation (Supplementary Figure 4).

### MPS2 Threshold Performance and Clinical Outcomes

Performance measures were calculated for MPS2 thresholds spanning 5%-15% (Supplementary Tables 1-2), and the threshold of 11.5% provided an optimal balance of biopsy avoidance and preserved GG≥2 cancer detection. Overall performance and clinical consequences of pre-biopsy testing with MPS2 and the PCPTrc were calculated per 1000 patients (Table 3). In the overall population, under a testing approach preserving detection of ≥92% of GG≥2 cancers, use of MPS2(BA) would have avoided 274 (36%) unnecessary biopsies and MPS2(BA+CF+PV) would have avoided 315 (42%) unnecessary biopsies, as compared to only 94 (13%) using the PCPTrc. In the repeat biopsy population, with both MPS2 and PCPTrc detecting 93% of GG≥2 cancers, MPS2 testing would have avoided 327-395 (44-53%) unnecessary biopsies as compared to only 20 (2.6%) unnecessary biopsies avoided using the PCPTrc. Similar findings were observed when limited to patients with PSA<10 ng/ml (Supplementary Table 3). Notably, only a single patient with GG≥3 cancer had a false negative MPS2 result. For GG≥3 cancer, the MPS2 models provided 98-100% NPV and 94-100% sensitivity across subgroups (Supplementary Table 4).

### SUBGROUP ANALYSIS: MPS2 + MRI

There were 47 patients that underwent pre-biopsy MRI (Supplementary Table 5). Among 19 patients (40%) with GG≥2 cancer, MPS2(BA) was positive in 18 (95%), while MRI was positive (PI-RADS≥3) in 15 (79%). Among seven cases where both MPS2(BA) and MRI were negative, zero (0%) had GG≥2 cancer. When both MPS2(BA) and MRI were positive (n=21), 14 (67%) had GG≥2 cancer. There were 19 patients with discordant MPS2(BA) and MRI findings, of which 5 (26%) had GG≥2. Full performance characteristics of MPS2 and MRI in this subgroup are provided in Supplementary Table 6.

## DISCUSSION

The MyProstateScore 2.0 test measures 18 cancer-associated and high-grade cancer-associated genes in urine to provide a percentage likelihood of detecting GG≥2 cancer on biopsy. In post-DRE urine, MPS2 was previously shown to improve detection of GG≥2 cancer relative to PSA, the PCPTrc, and two currently-available biomarker tests. While a practical strength of biomarker tests is their ease of use (e.g. tests can be readily sent from clinic), the need for DRE prior to urine collection precluded remote and at-home sample collection – an option increasingly sought in the expanding era of telehealth. Thus, the current study validated MPS2 using first-catch, non-DRE urine.

To provide flexibility for ordering clinicians, MPS2 can be performed without requiring any clinical data (MPS2-BA), with standard clinical data (MPS2-BA+CF), or with standard clinical data and prostate volume (MPS2-BA+CF+PV). Clinicians simply provide available data, and readily-interpretable results are provided based on the most informative model (Supplementary Figure 5). Importantly, the current analysis reveals that all MPS2 models provide substantial clinical improvement relative to the PCPTrc. In selecting patients for biopsy, under a clinical approach detecting 92% of GG≥2 cancers, use of MPS2 would have avoided 36-42% of unnecessary biopsies, as compared to 13% using the PCPTrc. Consistent with validation of the post-DRE assay,^12^ MPS2 outperformed the PCPTrc by a remarkable margin in the repeat biopsy setting. While both tests detected 93% of GG≥2 cancers, MPS2 allowed for avoidance of 44-53% of unnecessary biopsies as compared to only 2.6% using the PCPTrc. The drastic improvements observed in the repeat biopsy setting are unsurprising given the dependence of the PCPTrc on PSA, and the well-documented limitations of PSA in patients with a previous negative biopsy.^5^ By capturing 17 non-PSA, cancer-associated markers, the MPS2 test appears to provide uniquely strong clinical advantages in the repeat biopsy setting.

Based on the current analysis, the MPS2 non-DRE test appears to provide advantages relative to existing non-DRE urine tests. In the initial biopsy population, the MPS2 biomarker-only test (i.e. no clinical factors included) provided an AUC of 70%, in line with the 67-71% reported for the ExoDx Prostate Intelliscore (EPI) test,^24,26-27^ while the ability to include clinical data further improved the AUC of MPS2 to 76%. Acknowledging a limited sample size, the clinical improvements offered by MPS2 were particularly notable in patients with a previous negative biopsy. While MPS2(BA) allowed for avoidance of 44% of unnecessary biopsies (93% sensitivity, 44% specificity, 95% NPV), it is notable that prostate volume is available in nearly all men considering repeat biopsy, as volume is measured at the time of initial biopsy or by MRI, which is guideline-recommended prior to repeat biopsy.^5^ As such, the MPS2 model including prostate volume allowed for avoidance of 53% of unnecessary biopsies while missing only 6.8% of GG≥2 cancers (94% sensitivity, 53% specificity, 96% NPV). These findings compare very favorably to published EPI data using the validated threshold of 15.6, which allowed for avoidance of 27% of unnecessary biopsies while missing 18% of GG≥2 cancers (82% sensitivity, 27% specificity, 92% NPV), and findings using an adjusted threshold of 20 (82% sensitivity, 37% specificity, 94% NPV).^28^ While cross-study comparisons must be made with caution, and predictive values depend on cohort-specific prevalence, sensitivity and specificity are not cohort-dependent metrics.^11^ Finally, in contrast to the unitless number value returned by existing assays, MPS2 provides each patient’s individualized probability of detecting GG≥2 cancer. This allows for meaningful interpretation and discussion of risk, particularly in patients with results near the threshold value.

Biomarker tests and MRI have both been shown to increase specificity for higher-grade, clinically-significant cancers, and the optimal use of these tools has been explored in light of both performance and practicality. Several groups have described the merits of a diagnostic algorithm using biomarkers first – to effectively triage the number of men referred for MRI – followed by MRI to enhance the yield of biopsy. In addition to practical advantages, such an approach leverages the strengths of each test: the high rule-out ability of biomarkers, and the strong PPV and lesion-targeting of MRI. Initial data from the ProScreen trial, which used a 4-kallikrein biomarker test to select patients for MRI and possible biopsy, appear to support the proposed advantages of a biomarker-first testing approach.^10^ Compared to initial findings from the ERSPC,^29^ in which all men with elevated PSA underwent biopsy, ProScreen reduced the proportion of patients undergoing biopsy (3% vs. 22%) while achieving comparable detection of GG≥2 disease (1.7% vs. 1.8%). Promisingly, findings from our limited subgroup of patients with MPS2 and MRI suggest that combining these modalities provide strong rule-out and rule-in performance.

There are real and perceived limitations of the current study. First, only a limited proportion of patients underwent pre-biopsy MRI. As a result, the reference standard in most cases was systematic biopsy, which could result in undersampling and increase NPV relative to surgical pathology.^30^ At the same time, undersampling would reduce PPV, meaning that this limitation would have a balanced overall impact on test performance. Consistent with the ProScreen trial, the current analysis aimed to evaluate MPS2 as a first-line test after PSA to avoid the use of MRI and biopsy. Thus, data on the combined use of MPS2 and MRI were limited and may not reflect the overall at-risk population. Moreover, there were limited data describing the repeat biopsy population, African-American men, and patients with a suspicious DRE, and each of these subgroups merit further evaluation. These limitations notwithstanding, the current analysis demonstrates high clinical accuracy of the MPS2 urinary assay performed without DRE.

## CONCLUSIONS

The current study validated the 18-gene MPS2 assay in urine specimens obtained without DRE. In a clinically-pertinent testing population, MPS2 provided meaningful improvements in detection of GG≥2 cancer relative to PSA-based testing approaches. Importantly, improved diagnostic performance was observed in the baseline MPS2 model based on biomarker expression only, with further improvements observed with inclusion of optional clinical data. As such, MPS2 appears to provide a convenient, versatile, and highly accurate testing option to inform the need for MRI or biopsy in patients with elevated PSA.

## Supplementary Material

Supplementary Material

## Figures and Tables

**Table 1.** Demographic and clinical characteristics of study cohort

Characteristic	GG1/Benign	GG≥2	P-value
N (%)	163 (61)	103 (39)	
Median yrs age (IQR)	67 (61-71)	67 (64-72)	0.4
No. black race (%)	8 (4.9)	7 (6.8)	0.6
No. positive family history (%)	52 (32)	44 (43)	0.1
No. previous negative biopsy (%)	39 (24)	15 (15)	0.09
No. suspicious DRE (%)	5 (3.1)	13 (13)	0.004
Median ng/mL PSA (IQR)	6.4 (4.7-8.8)	6.8 (5.2-9.9)	0.060
Median prostate volume (IQR)	52 (40-69)	40 (31-55)	<0.001
Underwent MRI (%)	28 (17)	19 (18)	>0.9
PI-RADS 1-2 (%)	18 (11)	4 (3.9)	
PI-RADS 3 (%)	3 (1.8)	2 (1.9)	
PI-RADS 4 (%)	4 (2.5)	8 (7.8)	
PI-RADS 5 (%)	3 (1.8)	5 (4.9)	
Median MPS2 (BA) Score (IQR)	15% (7%-28%)	31% (18%-51%)	<0.001
Median MPS2 (BA+CF) Score (IQR)	15% (5%-28%)	36% (18%-65%)	<0.001
Median MPS2 (BA+CF+PV) Score (IQR)	13% (5%-28%)	41% (19%-72%)	<0.001

P-values calculated using Wilcoxon Rank-Sum test for medians and chi-squared or Fisher’s exact test for proportions.

Abbreviations: BA, Biomarkers Alone Model; BA+CF, Biomarkers plus Clinical Factors Model; BA+CF+PV, Biomarkers, Clinical Factors, and Prostate Volume Model; DRE, Digital Rectal Exam; GG, Grade Group.

**Table 2.** Discriminative accuracy of PSA, the PCPTrc, and MPS2 models for GG≥2 cancer

Model	AUC (95% CI)	P-value vs. PSA	P-value vs. PCPT	P-value vs. Preceding Model
PSA	57% (50%-64%)			
PCPTrc	62% (55%-69%)	0.035		
MPS2 (BA)	71% (65%-77%)	0.003	0.054	
MPS2 (BA+CF)	74% (68%-80%)	<0.001	0.004	0.076
MPS2 (BA+CF+PV)	77% (71%-83%)	<0.001	<0.001	0.01 (<0.001 vs. MPS2-BA)

Comparisons performed using the Delong method.

Abbreviations: BA, Biomarkers Alone Model; BA+CF, Biomarkers plus Clinical Factors Model; BA+CF+PV, Biomarkers, Clinical Factors, and Prostate Volume Model; DRE, Digital Rectal Exam; GG, Grade Group.

**Table 3.** Performance and clinical consequences of MPS2 and PCPTrc testing to select for biopsy per 1000 patients

Model	Population	Threshold	GG≥2 Detected (Sensitivity)	GG≥2 Missed (%)	Bx Avoided (%)	Unnec. Bx Avoided (Specificity)	NPV	PPV
MPS2 (BA)	Overall	11.5%	231 (92%)	19 (7.6%)	293 (29%)	274 (36%)	93%	33%
MPS2 (BA+CF)		11.5%	228 (91%)	22 (8.8%)	294 (29%)	272 (36%)	93%	32%
MPS2 (BA+CF+PV)		11.5%	235 (94%)	15 (6.0%)	330 (33%)	315 (42%)	95%	35%
PCPTrc		5.6%	230 (92%)	20 (8.0%)	114 (11%)	94 (13%)	83%	26%
MPS2 (BA)	Initial Bx	11.5%	230 (92%)	20 (8.0%)	280 (28%)	260 (35%)	93%	32%
MPS2 (BA+CF)		11.5%	227 (91%)	23 (9.2%)	281 (28%)	258 (34%)	92%	32%
MPS2 (BA+CF+PV)		11.5%	235 (94%)	15 (6.0%)	310 (31%)	295 (39%)	95%	34%
PCPTrc		6.3%	230 (92%)	20 (8.0%)	174 (20%)	154 (20%)	88%	28%
MPS2 (BA)	Repeat Bx	11.5%	233 (93%)	17 (6.8%)	344 (34%)	327 (44%)	95%	36%
MPS2 (BA+CF)		11.5%	233 (93%)	17 (6.8%)	344 (34%)	327 (44%)	95%	36%
MPS2 (BA+CF+PV)		11.5%	233 (93%)	17 (6.8%)	411 (41%)	395 (53%)	96%	40%
PCPTrc		4.5%	233 (93%)	17 (6.8%)	36 (3.6%)	20 (2.6%)	50%	27%

Abbreviations: BA, Biomarkers Alone Model; BA+CF, Biomarkers plus Clinical Factors Model; BA+CF+PV, Biomarkers, Clinical Factors, and Prostate Volume Model; DRE, Digital Rectal Exam; GG, Grade Group; NPV, Negative Predictive Value; PPV, Positive Predictive Value.

## Abbreviations and Acronyms

AUA: American Urological Association
AUC: Area Under the Curve
BA: Biomarkers Alone Model
BA+CF: Biomarkers plus Clinical Factors Model
BA+CF+PV: Biomarkers, Clinical Factors, and Prostate Volume Model
cDNA: Complementary DNA
Crt: Relative Cycle Threshold
DCA: Decision Curve Analysis
DRE: Digital Rectal Examination
EPI: ExoDx Prostate Intelliscore
ERSPC: European Randomized Study of Screening for Prostate Cancer
GG: Grade Group
KLK3: Kallikrein-Related Peptidase 3
MRI: Magnetic Resonance Imaging
MPS2: MyProstateScore 2.0
NPV: Negative Predictive Value
PCa: Prostate Cancer
PCPT: Prostate Cancer Prevention Trial
PI-RADS: Prostate Imaging Reporting and Data System
PPV: Positive Predictive Value
ProScreen: Prostate Cancer Screening Trial
PSA: Prostate-Specific Antigen
RNA: Ribonucleic Acid

## References

[1] Frånlund M, Månsson M, Godtman RA, Results from 22 years of followup in the Göteborg Randomized Population-Based Prostate Cancer Screening Trial. J Urol. 2022;208(2):292–300. doi:10.1097/JU.0000000000002696 35422134 PMC9275849

[2] Hendrick RE, Helvie MA. Mammography screening: A new estimate of number needed to screen to prevent one breast cancer death. AJR Am J Roentgenol. 2012;198(3):723–728. doi:10.2214/AJR.11.7146 22358016

[3] Fenton JJ, Weyrich MS, Durbin S, Liu Y, Bang H, Melnikow J. Prostate-specific antigen-based screening for prostate cancer: Evidence report and systematic review for the US Preventive Services Task Force. JAMA. 2018;319(18):1914–1931. doi:10.1001/jama.2018.3712 29801018

[4] National Comprehensive Cancer Network. Prostate cancer early detection (version 2.2024). Accessed July 31, 2024. https://www.nccn.org/professionals/physician_gls/pdf/prostate_detection.pdf

[5] Wei JT, Barocas D, Carlsson S, Early detection of prostate cancer: AUA/SUO Guideline Part I: Prostate Cancer Screening. J Urol. 2023;210(1):46–53. doi:10.1097/JU.0000000000003491 37096582 PMC11060750

[6] Hugosson J, Månsson M, Wallström J, Prostate Cancer Screening with PSA and MRI Followed by Targeted Biopsy Only. N Engl J Med. 2022;387(23):2126–2137. doi:10.1056/NEJMoa2209454 36477032 PMC9870590

[7] Leapman MS, Wang R, Park HS, Adoption of new risk stratification technologies within US hospital referral regions and association with prostate cancer management. JAMA Netw Open. 2021;4(10):e2128646.. doi:10.1001/jamanetworkopen.2021.28646 34623406 PMC8501394

[8] Sonn GA, Fan RE, Ghanouni P, Prostate magnetic resonance imaging interpretation varies substantially across radiologists. Eur Urol Focus. 2019;5(4):592–599. doi:10.1016/j.euf.2017.11.010 29226826

[9] Jiao B, Gulati R, Hendrix N, Economic evaluation of urine-based or magnetic resonance imaging reflex tests in men with intermediate prostate-specific antigen levels in the United States. Value Health. 2021;24(8):1111–1117. doi:10.1016/j.jval.2021.02.009 34372976 PMC8358184

[10] Auvinen A, Tammela TLJ, Mirtti T, Prostate cancer screening with PSA, kallikrein panel, and MRI: The ProScreen randomized trial. JAMA. 2024;331(17):1452–1459. doi:10.1001/jama.2024.3841 38581254 PMC10999002

[11] Eyrich NW, Morgan TM, Tosoian JJ. Biomarkers for detection of clinically significant prostate cancer: Contemporary clinical data and future directions. Transl Androl Urol. 2021;10(7):3091–3103. doi:10.21037/tau-20-1151 34430413 PMC8350244

[12] Tosoian JJ, Zhang Y, Xiao L, Development and validation of an 18-gene urine test for high-grade prostate cancer. JAMA Oncol. 2024;10(6):726–736. doi:10.1001/jamaoncol.2024.0455 38635241 PMC11190811

[13] Thompson IM, Ankerst DP, Chi C, Assessing prostate cancer risk: Results from the Prostate Cancer Prevention Trial. J Natl Cancer Inst. 2006;98(8):529–534. doi:10.1093/jnci/djj131 16622122

[14] DeLong ER, DeLong DM, Clarke-Pearson DL. Comparing the areas under two or more correlated receiver operating characteristic curves: A nonparametric approach. Biometrics. 1988;44(3):837–845. 3203132

[15] Vickers AJ, Van Calster B, Steyerberg EW. Net benefit approaches to the evaluation of prediction models, molecular markers, and diagnostic tests. BMJ. 2016;352:i6. doi:10.1136/bmj.i6 26810254 PMC4724785

[16] Vickers AJ, Elkin EB. Decision curve analysis: A novel method for evaluating prediction models. Med Decis Making. 2006;26(6):565–574. doi:10.1177/0272989X06295361 17099194 PMC2577036

[17] Morrow DA, Cook NR. Determining decision limits for new biomarkers: Clinical and statistical considerations. Clin Chem. 2011;57(1):1–3. doi:10.1373/clinchem.2010.155879 21078839

[18] Morrow DA, Cannon CP, Jesse RL, National Academy of Clinical Biochemistry Laboratory Medicine Practice Guidelines: Clinical characteristics and utilization of biochemical markers in acute coronary syndromes. Circulation. 2007;115(13):e356–e375. doi:10.1161/CIRCULATIONAHA.107.182882 17384331

[19] Tosoian JJ, Trock BJ, Morgan TM, Use of the MyProstateScore test to rule out clinically significant cancer: Validation of a straightforward clinical testing approach. J Urol. 2021;205(3):732–739. doi:10.1097/JU.0000000000001430 33080150 PMC8189629

[20] Centers for Medicare & Medicaid Services. Biomarker testing for prostate cancer diagnosis. CMS.gov. Updated March 1, 2024. Accessed July 30, 2024. https://www.cms.gov/medicare-coverage-database/view/lcd.aspx?LCDId=37733.

[21] Parekh DJ, Punnen S, Sjoberg DD, A multi-institutional prospective trial in the USA confirms that the 4Kscore accurately identifies men with high-grade prostate cancer. Eur Urol. 2015;68(3):464–470. doi:10.1016/j.eururo.2014.10.021 25454615

[22] Loeb S, Sanda MG, Broyles DL, The prostate health index selectively identifies clinically significant prostate cancer. J Urol. 2015;193(4):1163–1169. doi:10.1016/j.juro.2014.10.121 25463993 PMC4404198

[23] de la Calle C, Patil D, Wei JT, Multicenter evaluation of the Prostate Health Index to detect aggressive prostate cancer in biopsy naïve men. J Urol. 2015;194(1):65–72. doi:10.1016/j.juro.2015.01.091 25636659 PMC4696043

[24] McKiernan J, Donovan MJ, O'Neill V, A novel urine exosome gene expression assay to predict high-grade prostate cancer at initial biopsy. JAMA Oncol. 2016;2(7):882–889. doi:10.1001/jamaoncol.2016.0097 27032035

[25] Van Neste L, Henao R, Wojno KJ, Development and optimization of a subtraction-normalized immunocyte profiling signature for prostate cancer active surveillance risk stratification. J Urol. 2024;211(3):415–425. doi:10.1097/JU.0000000000003824 38147400 PMC12721612

[26] McKiernan J, Donovan MJ, Margolis E, A Prospective Adaptive Utility Trial to Validate Performance of a Novel Urine Exosome Gene Expression Assay to Predict High-grade Prostate Cancer in Patients with Prostate-specific Antigen 2-10ng/ml at Initial Biopsy. Eur Urol. 2018;74(6):731–738. doi:10.1016/j.eururo.2018.08.019 30237023

[27] Kretschmer A, Kajau H, Margolis E, Validation of a CE-IVD, urine exosomal RNA expression assay for risk assessment of prostate cancer prior to biopsy. Sci Rep. 2022;12(1):4777. Published 2022 Mar 21. doi:10.1038/s41598-022-08608-z 35314720 PMC8938406

[28] McKiernan J, Noerholm M, Tadigotla V, A urine-based exosomal gene expression test stratifies risk of high-grade prostate cancer in men with prior negative prostate biopsy undergoing repeat biopsy. BMC Urol. 2020;20(1):138. doi:10.1186/s12894-020-00712-4 32873277 PMC7466797

[29] van der Cruijsen-Koeter IW, Roobol MJ, Wildhagen MF, van der Kwast TH, Kirkels WJ, Schröder FH. Tumor characteristics and prognostic factors in two subsequent screening rounds with four-year interval within prostate cancer screening trial, ERSPC Rotterdam. Urology. 2006;68(3):615–620. doi:10.1016/j.urology.2006.03.015 17010732

[30] Epstein JI, Feng Z, Trock BJ, Pierorazio PM. Upgrading and downgrading of prostate cancer from biopsy to radical prostatectomy: Incidence and predictive factors using the modified Gleason grading system and factoring in tertiary grades. Eur Urol. 2012;61(5):1019–1024. doi:10.1016/j.eururo.2012.01.050 22336380 PMC4659370

