# Supplementary material for: Clinical Validation of MyProstateScore 2.0 Testing Using First-Catch, Non-DRE Urine
Source: J Urol. Author manuscript; Available in PMC 2025 May 14. (PMC11981841; doi:10.1097/JU.0000000000004421)
Supplement: Supplementary Material [file NIHMS2047663-supplement-Supplementary_Material.docx]

# SUPPLemeNTAry TABLES

**Supplementary Table 1.** *Performance of MPS2 threshold values in patients undergoing initial biopsy*

| Model | Threshold | Sensitivity | Specificity | NPV | PPV |
| --- | --- | --- | --- | --- | --- |
| MPS2 (BA) | 5.0% | 97% | 13% | 92% | 27% |
|  | 5.5% | 95% | 15% | 91% | 27% |
|  | 6.0% | 95% | 18% | 92% | 28% |
|  | 6.5% | 95% | 19% | 92% | 28% |
|  | 7.0% | 95% | 20% | 93% | 28% |
|  | 7.5% | 95% | 22% | 93% | 29% |
|  | 8.0% | 95% | 26% | 94% | 30% |
|  | 8.5% | 95% | 28% | 95% | 31% |
|  | 9.0% | 94% | 29% | 94% | 31% |
|  | 9.5% | 94% | 31% | 94% | 31% |
|  | 10.0% | 94% | 31% | 94% | 31% |
|  | 10.5% | 94% | 33% | 95% | 32% |
|  | 11.0% | 92% | 34% | 93% | 32% |
|  | 11.5% | 92% | 35% | 93% | 32% |
|  | 12.0% | 91% | 36% | 92% | 32% |
|  | 12.5% | 90% | 37% | 92% | 32% |
|  | 13.0% | 89% | 38% | 91% | 32% |
|  | 13.5% | 88% | 39% | 90% | 32% |
|  | 14.0% | 88% | 40% | 90% | 33% |
|  | 14.5% | 88% | 40% | 91% | 33% |
|  | 15.0% | 85% | 40% | 89% | 32% |
| MPS2 (BA+CF) | 5.0% | 95% | 15% | 90% | 27% |
|  | 5.5% | 94% | 16% | 89% | 27% |
|  | 6.0% | 94% | 18% | 90% | 28% |
|  | 6.5% | 94% | 20% | 91% | 28% |
|  | 7.0% | 94% | 22% | 92% | 29% |
|  | 7.5% | 94% | 24% | 92% | 29% |
|  | 8.0% | 94% | 28% | 93% | 30% |
|  | 8.5% | 93% | 30% | 93% | 31% |
|  | 9.0% | 93% | 31% | 93% | 31% |
|  | 9.5% | 93% | 32% | 93% | 31% |
|  | 10.0% | 93% | 33% | 93% | 32% |
|  | 10.5% | 92% | 34% | 93% | 32% |
|  | 11.0% | 92% | 34% | 93% | 32% |
|  | 11.5% | 91% | 34% | 92% | 32% |
|  | 12.0% | 90% | 36% | 91% | 32% |
|  | 12.5% | 87% | 36% | 89% | 31% |
|  | 13.0% | 87% | 37% | 90% | 32% |
|  | 13.5% | 84% | 38% | 87% | 31% |
|  | 14.0% | 84% | 39% | 88% | 32% |
|  | 14.5% | 83% | 39% | 87% | 31% |
|  | 15.0% | 83% | 40% | 87% | 32% |
| MPS2 (BA+CF+PV) | 5.0% | 98% | 16% | 95% | 28% |
|  | 5.5% | 98% | 16% | 95% | 28% |
|  | 6.0% | 98% | 20% | 96% | 29% |
|  | 6.5% | 98% | 23% | 97% | 30% |
|  | 7.0% | 97% | 25% | 96% | 30% |
|  | 7.5% | 95% | 29% | 95% | 31% |
|  | 8.0% | 95% | 30% | 95% | 31% |
|  | 8.5% | 95% | 30% | 95% | 31% |
|  | 9.0% | 95% | 31% | 95% | 32% |
|  | 9.5% | 95% | 32% | 95% | 32% |
|  | 10.0% | 95% | 35% | 96% | 33% |
|  | 10.5% | 94% | 38% | 95% | 34% |
|  | 11.0% | 94% | 39% | 95% | 34% |
|  | 11.5% | 94% | 39% | 95% | 34% |
|  | 12.0% | 94% | 40% | 95% | 34% |
|  | 12.5% | 94% | 40% | 95% | 34% |
|  | 13.0% | 93% | 41% | 95% | 34% |
|  | 13.5% | 92% | 41% | 94% | 34% |
|  | 14.0% | 92% | 41% | 94% | 34% |
|  | 14.5% | 92% | 42% | 94% | 34% |
|  | 15.0% | 88% | 42% | 92% | 34% |

Abbreviations: BA, Biomarkers Alone Model; BA+CF, Biomarkers plus Clinical Factors Model; BA+CF+PV, Biomarkers, Clinical Factors, and Prostate Volume Model; NPV, Negative Predictive Value; PPV, Positive Predictive Value.

**Supplementary Table 2.** *Performance of MPS2 threshold values in patients undergoing repeat biopsy*

| Model | Threshold | Sensitivity | Specificity | NPV | PPV |
| --- | --- | --- | --- | --- | --- |
| MPS2 (BA) | 5.0% | 100% | 10% | 100% | 27% |
|  | 5.5% | 100% | 13% | 100% | 28% |
|  | 6.0% | 100% | 18% | 100% | 29% |
|  | 6.5% | 100% | 18% | 100% | 29% |
|  | 7.0% | 100% | 21% | 100% | 30% |
|  | 7.5% | 100% | 23% | 100% | 30% |
|  | 8.0% | 100% | 26% | 100% | 31% |
|  | 8.5% | 100% | 26% | 100% | 31% |
|  | 9.0% | 100% | 28% | 100% | 32% |
|  | 9.5% | 100% | 31% | 100% | 33% |
|  | 10.0% | 93% | 33% | 94% | 32% |
|  | 10.5% | 93% | 38% | 95% | 34% |
|  | 11.0% | 93% | 41% | 95% | 35% |
|  | 11.5% | 93% | 44% | 95% | 36% |
|  | 12.0% | 93% | 44% | 95% | 36% |
|  | 12.5% | 93% | 46% | 95% | 37% |
|  | 13.0% | 93% | 46% | 95% | 37% |
|  | 13.5% | 93% | 46% | 95% | 37% |
|  | 14.0% | 87% | 46% | 91% | 35% |
|  | 14.5% | 87% | 46% | 91% | 35% |
|  | 15.0% | 87% | 46% | 91% | 35% |
| MPS2 (BA+CF)* | 5.0% | 100% | 10% | 100% | 27% |
|  | 5.5% | 100% | 13% | 100% | 28% |
|  | 6.0% | 100% | 18% | 100% | 29% |
|  | 6.5% | 100% | 18% | 100% | 29% |
|  | 7.0% | 100% | 21% | 100% | 30% |
|  | 7.5% | 100% | 23% | 100% | 30% |
|  | 8.0% | 100% | 26% | 100% | 31% |
|  | 8.5% | 100% | 26% | 100% | 31% |
|  | 9.0% | 100% | 28% | 100% | 32% |
|  | 9.5% | 100% | 31% | 100% | 33% |
|  | 10.0% | 93% | 33% | 94% | 32% |
|  | 10.5% | 93% | 38% | 95% | 34% |
|  | 11.0% | 93% | 41% | 95% | 35% |
|  | 11.5% | 93% | 44% | 95% | 36% |
|  | 12.0% | 93% | 44% | 95% | 36% |
|  | 12.5% | 93% | 46% | 95% | 37% |
|  | 13.0% | 93% | 46% | 95% | 37% |
|  | 13.5% | 93% | 46% | 95% | 37% |
|  | 14.0% | 87% | 46% | 91% | 35% |
|  | 14.5% | 87% | 46% | 91% | 35% |
|  | 15.0% | 87% | 46% | 91% | 35% |
| MPS2 (BA+CF+PV) | 5.0% | 93% | 29% | 93% | 30% |
|  | 5.5% | 93% | 34% | 94% | 32% |
|  | 6.0% | 93% | 37% | 94% | 33% |
|  | 6.5% | 93% | 37% | 94% | 33% |
|  | 7.0% | 93% | 39% | 95% | 34% |
|  | 7.5% | 93% | 42% | 95% | 35% |
|  | 8.0% | 93% | 42% | 95% | 35% |
|  | 8.5% | 93% | 45% | 95% | 36% |
|  | 9.0% | 93% | 47% | 96% | 37% |
|  | 9.5% | 93% | 47% | 96% | 37% |
|  | 10.0% | 93% | 47% | 96% | 37% |
|  | 10.5% | 93% | 50% | 96% | 38% |
|  | 11.0% | 93% | 50% | 96% | 38% |
|  | 11.5% | 93% | 53% | 96% | 40% |
|  | 12.0% | 80% | 53% | 89% | 36% |
|  | 12.5% | 80% | 55% | 89% | 37% |
|  | 13.0% | 80% | 55% | 89% | 37% |
|  | 13.5% | 80% | 58% | 90% | 39% |
|  | 14.0% | 80% | 58% | 90% | 39% |
|  | 14.5% | 80% | 58% | 90% | 39% |
|  | 15.0% | 80% | 61% | 90% | 40% |

Abbreviations: BA, Biomarkers Alone Model; BA+CF, Biomarkers plus Clinical Factors Model; BA+CF+PV, Biomarkers, Clinical Factors, and Prostate Volume Model; NPV, Negative Predictive Value; PPV, Positive Predictive Value. *As described in the text, addition of clinical factors did not improve model performance in the repeat biopsy population, thus the MPS2(BA) model is used in the BA and BA+CF settings.

**Supplementary Table 3.** *MPS2 performance measures for GG≥2 cancer at threshold value 11.5% in the subgroup of patients with PSA<10 ng/ml*

| Model | Population | Sensitivity | Specificity | NPV | PPV |
| --- | --- | --- | --- | --- | --- |
| MPS2 (BA) | Overall | 91% | 35% | 92% | 32% |
| MPS2 (BA+CF) | N=211 | 88% | 35% | 90% | 31% |
| MPS2 (BA+CF+PV) |  | 92% | 41% | 94% | 34% |
| MPS2 (BA) | Initial Bx | 91% | 35% | 92% | 32% |
| MPS2 (BA+CF) | N=178 | 88% | 35% | 90% | 31% |
| MPS2 (BA+CF+PV) |  | 92% | 40% | 94% | 34% |
| MPS2 (BA) | Repeat Bx | 91% | 36% | 92% | 32% |
| MPS2 (BA+CF) | N=33 | 91% | 36% | 92% | 32% |
| MPS2 (BA+CF+PV) |  | 91% | 46% | 94% | 36% |

Abbreviations: BA, Biomarkers Alone Model; BA+CF, Biomarkers plus Clinical Factors Model; BA+CF+PV, Biomarkers, Clinical Factors, and Prostate Volume Model; NPV, Negative Predictive Value; PPV, Positive Predictive Value.

**Supplementary Table 4.** *MPS2 performance measures for GG≥3 cancer at threshold value 11.5%*

| Model | Population | Sensitivity | Specificity | NPV | PPV |
| --- | --- | --- | --- | --- | --- |
| MPS2 (BA) | Overall | 95% | 37% | 98% | 16% |
| MPS2 (BA+CF) | N=266 | 95% | 41% | 98% | 17% |
| MPS2 (BA+CF+PV) |  | 95% | 43% | 99% | 17% |
| MPS2 (BA) | Initial Bx | 94% | 35% | 98% | 17% |
| MPS2 (BA+CF) | N=212 | 94% | 34% | 98% | 17% |
| MPS2 (BA+CF+PV) |  | 94% | 40% | 98% | 18% |
| MPS2 (BA) | Repeat Bx | 100% | 44% | 100% | 12% |
| MPS2 (BA+CF) | N=54 | 100% | 44% | 100% | 12% |
| MPS2 (BA+CF+PV) |  | 100% | 53% | 100% | 14% |

Abbreviations: BA, Biomarkers Alone Model; BA+CF, Biomarkers plus Clinical Factors Model; BA+CF+PV, Biomarkers, Clinical Factors, and Prostate Volume Model; NPV, Negative Predictive Value; PPV, Positive Predictive Value.

**Supplementary Table 5.** *Demographic and clinical characteristics of the subgroup with MRI (N=47)*

| Characteristic | GG1/Benign | GG≥2 | P-value |
| --- | --- | --- | --- |
| N | 28 (60%) | 19 (40%) | N/A |
| Median yrs age (IQR) | 70 (64-72) | 70 (64-73) | >0.9 |
| No. black race (%) | 0 (0%) | 1 (5%) | 0.4 |
| No. positive family history (%) | 12 (43%) | 8 (42%) | >0.9 |
| No. previous negative biopsy (%) | 21 (75%) | 7 (37%) | 0.01 |
| No. suspicious DRE (%) | 0 (0%) | 1 (5%) | 0.4 |
| Median ng/mL PSA (IQR) | 7.8 (6-11) | 6.8 (4.8-11) | 0.6 |
| Median prostate volume (IQR) | 61 (43-84) | 48 (41-60) | 0.2 |
| PI-RADS 1-2 (%) | 18 (64%) | 4 (21%) | 0.01 |
| PI-RADS 3 (%) | 3 (11%) | 2 (11%) | >0.9 |
| PI-RADS 4 (%) | 4 (14%) | 8 (42%) | 0.04 |
| PI-RADS 5 (%) | 3 (11%) | 5 (26%) | 0.2 |
| Median MPS2 (BA) Value (IQR) | 16% (7%-35%) | 33% (24%-43%) | 0.03 |
| Median MPS2 (BA+CF) Value (IQR) | 16% (4%-22%) | 32% (21%-66%) | <0.001 |
| Median MPS2 (BA+CF+PV) Value (IQR) | 13% (4%-24%) | 35% (25%-61%) | <0.001 |

P-values calculated using Wilcoxon Rank-Sum test for medians and chi-squared or Fisher’s exact test for proportions.

Abbreviations: BA, Biomarkers Alone Model; BA+CF, Biomarkers plus Clinical Factors Model; BA+CF+PV, Biomarkers, Clinical Factors, and Prostate Volume Model; DRE, Digital Rectal Exam; GG, Grade Group.

**Supplementary Table 6.** *Clinical performance of MPS2 Models and MRI in the MRI subgroup (N=47)*

| **Model** | **Threshold** | **Sensitivity** | **Specificity** | **NPV** | **PPV** |
| --- | --- | --- | --- | --- | --- |
| MPS2 (BA) | 11.5% | 95% | 36% | 91% | 50% |
| MPS2 (BA+CF) | 11.5% | 95% | 37% | 91% | 51% |
| MPS2 (BA+CF+PV) | 11.5% | 95% | 41% | 92% | 53% |
| MRI | PI-RADS≥3 | 79% | 64% | 82% | 60% |
| MRI | PI-RADS≥4 | 68% | 75% | 78% | 65% |

Abbreviations: BA, Biomarkers Alone Model; BA+CF, Biomarkers plus Clinical Factors Model; BA+CF+PV, Biomarkers, Clinical Factors, and Prostate Volume Model; NPV, Negative Predictive Value; PPV, Positive Predictive Value.

# SUPPLeMENTAry FIGURES


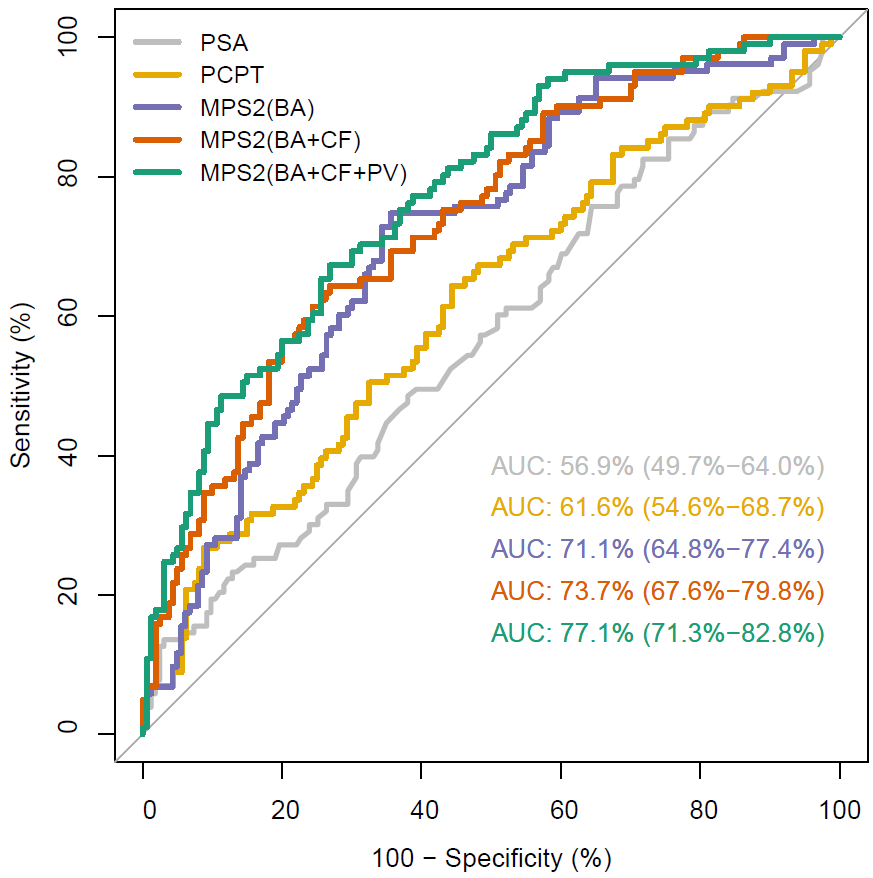


**Supplementary Figure 1.** Receiver-operating characteristic (ROC) curves with corresponding areas under the curve (AUC) and 95% confidence intervals for PSA, the PCPT risk calculator, and MPS2 models. BA, Biomarkers Alone Model; BA+CF, Biomarkers plus Clinical Factors Model; BA+CF+PV, Biomarkers, Clinical Factors, and Prostate Volume Model.


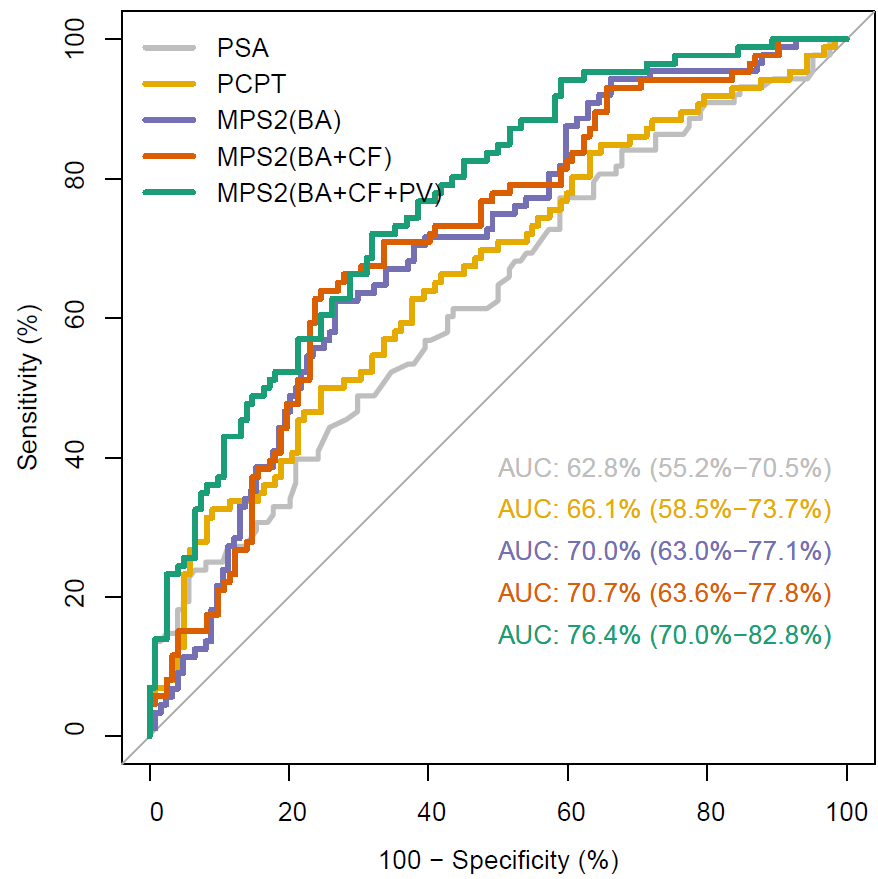


**Supplementary Figure 2.** Receiver-operating characteristic (ROC) curves with corresponding areas under the curve (AUC) and 95% confidence intervals for PSA, the PCPT risk calculator, and MPS2 models in the biopsy-naïve subpopulation. BA, Biomarkers Alone Model; BA+CF, Biomarkers plus Clinical Factors Model; BA+CF+PV, Biomarkers, Clinical Factors, and Prostate Volume Model.


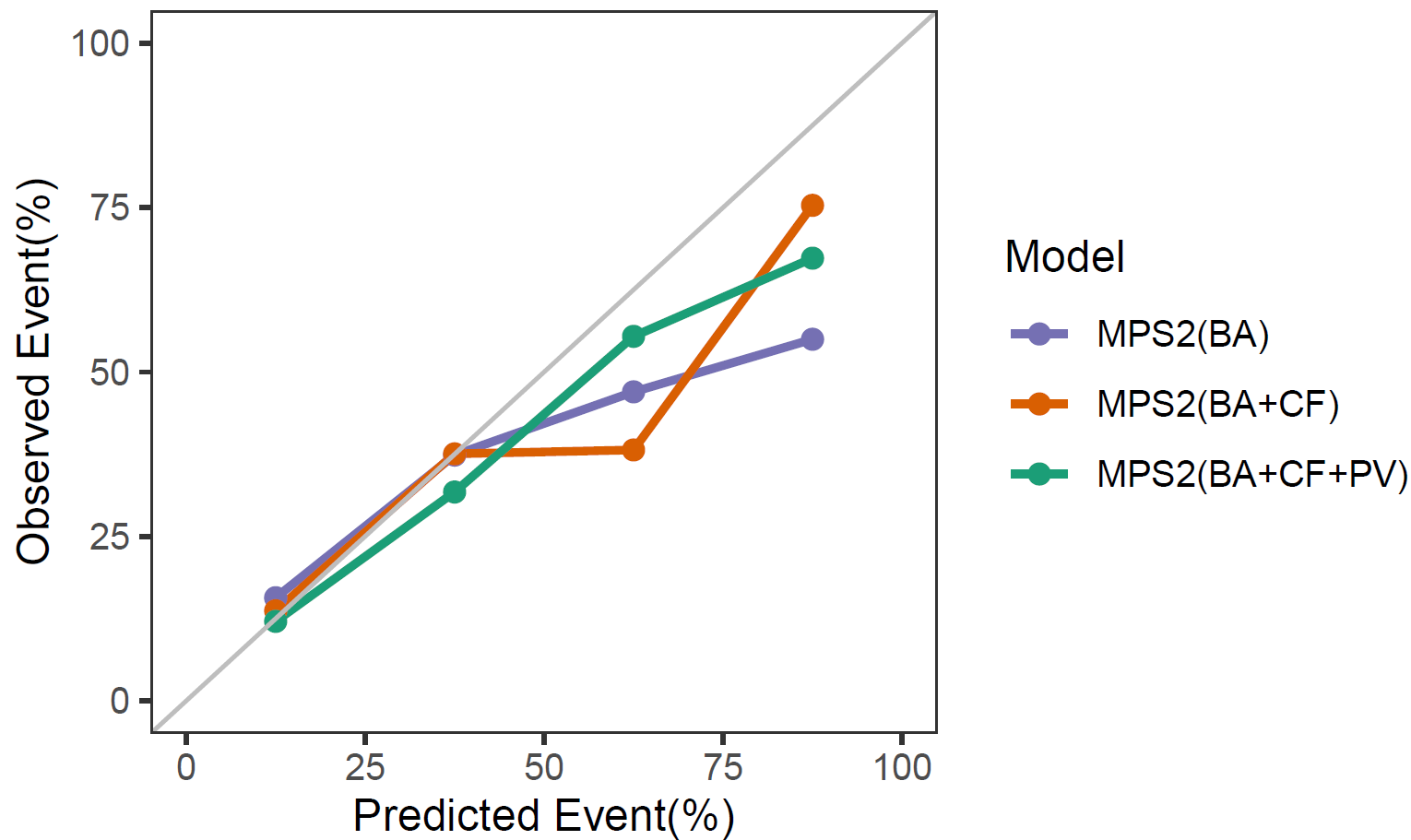


**Supplementary Figure 3.** Calibration curves for GG≥2 cancer for MPS2 models in a re-sampled validation cohort with 25% prevalence of GG≥2 cancer, consistent with published biomarker validation populations (17%-31%).^21-25^ BA, Biomarkers Alone Model; BA+CF, Biomarkers plus Clinical Factors Model; BA+CF+PV, Biomarkers, Clinical Factors, and Prostate Volume Model.


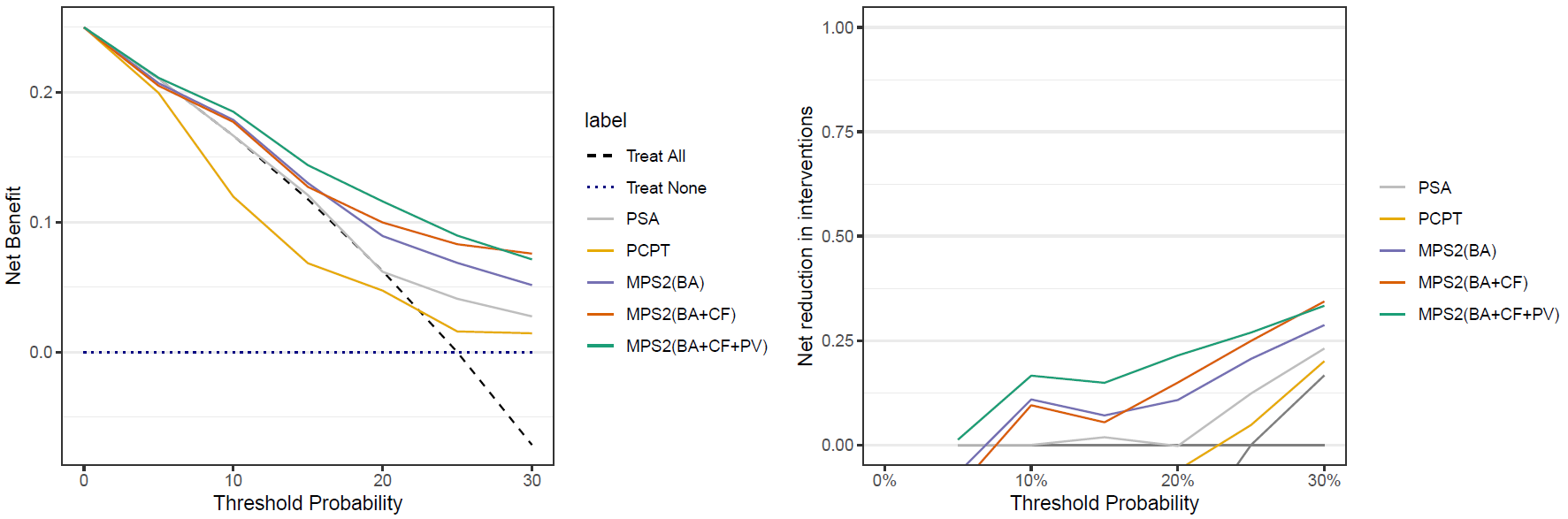


**Supplementary Figure 4.** Decision curve analysis (DCA) plots for the outcome of GG≥2 cancer based on pre-biopsy testing with PSA, the PCPT risk calculator, and MPS2 models in the biopsy-naïve population compared to baseline approaches of biopsying all patients and biopsying no patients. BA, Biomarkers Alone Model; BA+CF, Biomarkers plus Clinical Factors Model; BA+CF+PV, Biomarkers, Clinical Factors, and Prostate Volume Model.

**(A)** Net clinical benefit, in which the unit of net benefit (y-axis) is true positives. A net benefit of 0.1 is equivalent to an approach in which an additional 10 patients per 100 are directed to biopsy, and all 10 patients are found to have GG≥2 cancer.
**(B)** Net reduction in biopsies, in which the y-axis represents the net reduction in biopsies performed per 100 patients without missing a single diagnosis of GG≥2 cancer.

**
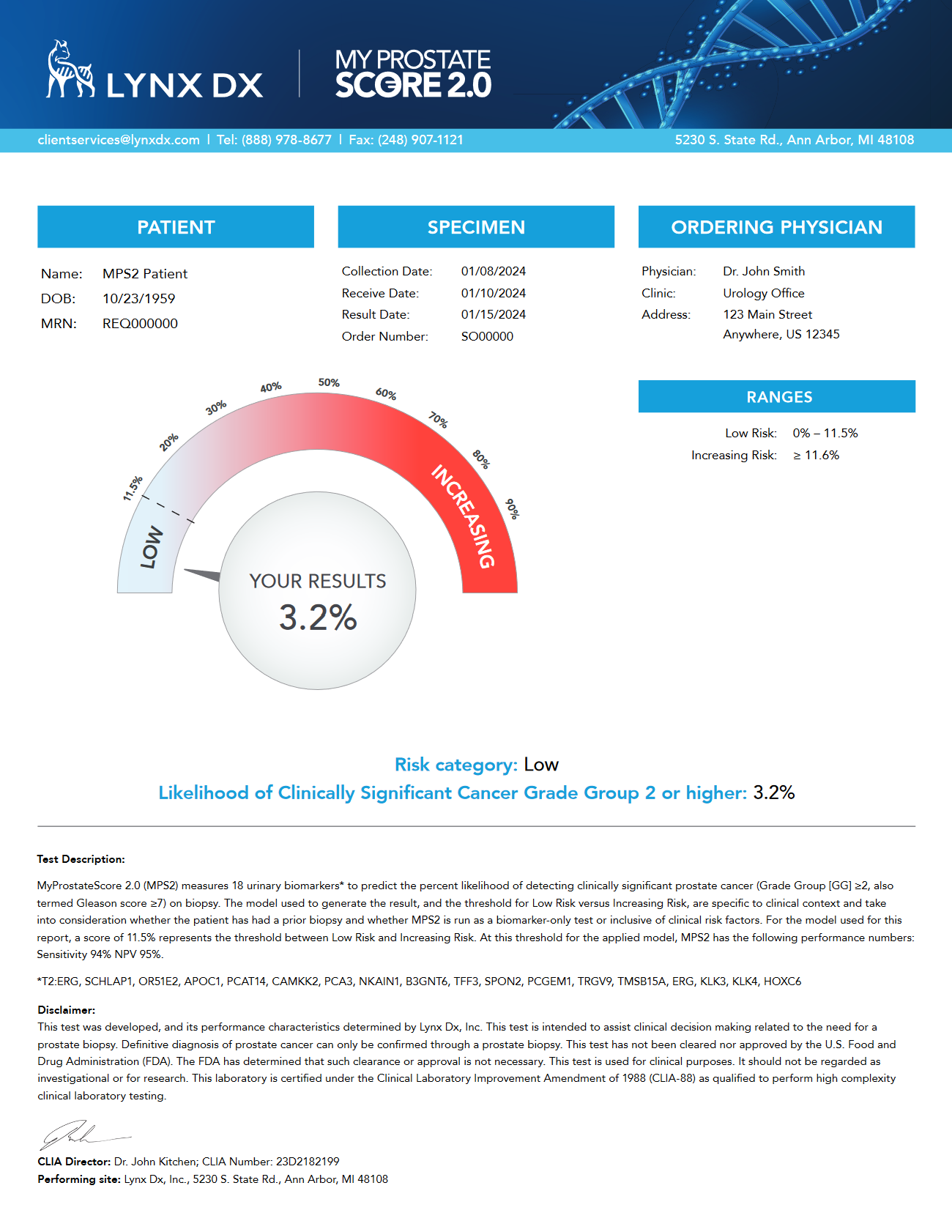
**

**Supplementary Figure 5.** Example MPS2 result report.
